# Estimation of epidemiological parameters and ascertainment rate from early transmission of COVID-19 across Africa

**DOI:** 10.1098/rsos.230316

**Published:** 2023-09-20

**Authors:** Qing Han, Nicola Bragazzi, Ali Asgary, James Orbinski, Jianhong Wu, Jude Dzevela Kong

**Affiliations:** ^1^ Africa-Canada Artificial Intelligence and Data Innovation Consortium (ACADIC), York University, Keele Campus, 4700 Keele Street, Toronto, Ontario, Canada M3J 1P3; ^2^ Department of Mathematics and Statistics, Laboratory for Industrial and Applied Mathematics (LIAM), York University, Keele Campus, 4700 Keele Street, Toronto, Ontario, Canada M3J 1P3; ^3^ Disaster and Emergency Management, School of Administrative Studies, Faculty of Liberal Arts and Professional Studies, York University, Keele Campus, 4700 Keele Street, Toronto, Ontario, Canada M3J 1P3; ^4^ Dahdaleh Institute for Global Health Research, York University, Keele Campus, 4700 Keele Street, Toronto, Ontario, Canada M3J 1P3

**Keywords:** COVID-19, ascertainment rate, under-reporting, mathematical model, Africa

## Abstract

Country reported case counts suggested a slow spread of SARS-CoV-2 in the initial phase of the COVID-19 pandemic in Africa. Owing to inadequate public awareness, unestablished monitoring practices, limited testing and stigmas, there might exist extensive under-ascertainment of the true number of cases, especially at the beginning of the novel epidemic. We developed a compartmentalized epidemiological model to track the early epidemics in 54 African countries. Data on the reported cumulative number of cases and daily confirmed cases were used to fit the model for the time period with no or little massive national interventions yet in each country. We estimated that the mean basic reproduction number is 2.02 (s.d. 0.7), with a range between 1.12 (Zambia) and 3.64 (Nigeria). The mean overall report rate was estimated to be 5.37% (s.d. 5.71%), with the highest 30.41% in Libya and the lowest 0.02% in São Tomé and Príncipe. An average of 5.46% (s.d. 6.4%) of all infected cases were severe cases and 66.74% (s.d. 17.28%) were asymptomatic ones. The estimated low reporting rates in Africa suggested a clear need for improved reporting and surveillance systems in these countries.

## 1. Introduction

The still ongoing coronavirus disease 2019 (COVID-19) pandemic, caused by the severe acute respiratory syndrome-related coronavirus type 2 (SARS-CoV-2) [1], first emerged in late December 2019 in the city of Wuhan, province of Hubei, mainland China [2]. The virus rapidly spread to the neighbouring cities and provinces of China, and thereafter was reported in Europe, America, as well as in other continents. As a result, the Director-General of the World Health Organization (WHO) declared COVID-19 initially as a public health emergency of international concern (PHEIC) on 30 January 2021 [3], and subsequently, as a global pandemic on 11 March 2020 [4]. As of 5 May 2022, the COVID-19 pandemic is affecting more than 220 countries and territories, with a dramatically high toll of infections (more than 513 million cases and six million deaths) [5].

However, the COVID-19 spread has been, and still is, quite uneven. Since the report of the first case of COVID-19 in Egypt on 14 February 2020 [6], the increase of new infections in African countries has been relatively modest compared to the rest of the globe, suggesting a slow spread of SARS-CoV-2 in Africa [7–9]. By 5 May 2022, 47 countries in the WHO African region had been affected with more than eight million cases and over 170 000 deaths [10], significantly lower compared to over 216 million cases and 1.9 million deaths in Europe, 153 million cases and 2.7 million deaths in the Americas and 57 million cases and 700 000 deaths in South-East Asia [5]. Some features of SARS-CoV-2 itself make it challenging for case detection including the existence of asymptomatic infections which are nonetheless capable of transmitting the pathogen [11,12]. Moreover, research suggested that a smaller fraction of clinical symptoms are manifested among the young population [13]. Therefore for countries with younger age structures like those in Africa (life expectancy of sub-Saharan Africa in 2019 is 61.63 years, compared to 75.40 years for East Asia, 73.94 for Europe and Central Asia and 73.78 for North America and Middle East [14]), more cases tend to go undetected. Nevertheless, given limited testing and public health resources, inadequate public awareness, cultural stigmatization, self-medication and the use of complementary/alternative medicine, or a not yet well established monitoring practice in the initial outbreak on the continent, the true number of cases might have been largely under-estimated. According to WHO, only one in seven cases was being detected in Africa [15].

The COVID-19 transmission in Africa has been studied in several aspects: the basic reproduction number in the initial phase was estimated for selected countries in Africa using a mechanistic model with a Bayesian inference framework [16], phenomenological models [17], and exponential growth rate [18], time-varying effective reproduction number and infection attack rate were also estimated [19], the effect of different lockdown and control strategies on COVID-19 transmission in African and West African countries was assessed [20,21], the spatio-temporal dynamics of COVID-19 within the first 62 days on the continent [22] was investigated and the preparedness and vulnerability of African countries against imported cases were evaluated [23]. There have also been estimations of under-ascertainment of COVID-19 infections in many locations around the world by various methods [24–27]. However, estimation and description for and among African countries remain lacking.

In this study, we reconstructed the initial transmission of SARS-CoV-2 in 54 African countries when there are no major mitigation interventions using a deterministic mathematical model accounting for under-reporting and also various levels of severity of infections. Reporting rates and critical quantities characterizing COVID-19 transmission within each country were estimated which constitute an epidemic profile for Africa for the initial stage of COVID-19.

## 2. Material and methods

### 2.1. Mathematical model

**Figure 1.**
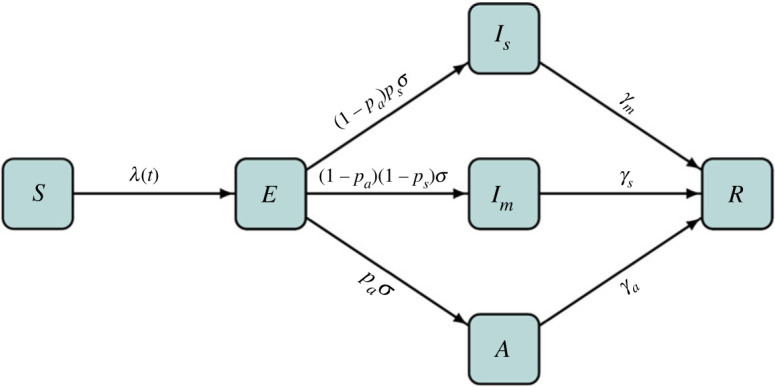
An illustration of the compartmentalized mathematical model for the early transmission of COVID-19.

The dynamics of COVID-19 in the early stage when major mitigation measures were not in place can be characterized by a simple susceptible–exposed–infectious–recovered (SEIR) model with the infected state further stratified into three compartments: severely infected (*I*_*s*_), mildly infected (*I*_*m*_) and asymptomatically infected (*A*). The mathematical model we developed to describe the initial dynamics of COVID-19 with under-reporting is thus based on the augmented SEIR model presented in (2.1) with the corresponding illustration diagram depicted in figure 1.

2.1 $$\begin{array}{rl} & \frac{\;dS}{\;dt}=-\beta \frac{\rho_{s}I_{s}+I_{m}+\rho_{a}A}{N}S,\\ & \frac{\;dE}{\;dt}=\beta \frac{\rho_{s}I_{s}+I_{m}+\rho_{a}A}{N}S-\sigma E,\\ & \frac{\;dI_{s}}{\;dt}=(1-p_{a})p_{s}\sigma E-\gamma_{s}I_{s},\\ & \frac{\;dI_{m}}{\;dt}=(1-p_{a})(1-p_{s})\sigma E-\gamma_{m}I_{m},\\ & \frac{\;dA}{\;dt}=p_{a}\sigma E-\gamma_{a}A\\ \;\mathrm{and}\; & \frac{\;dR}{\;dt}=\gamma_{s}I_{s}+\gamma_{m}I_{m}+\gamma_{a}A.\; \end{array}\}$$

In (2.1), *S*, *E*, *I*_*s*_, *I*_*m*_, *A* and *R* are numbers of susceptible, exposed, severely infected, mildly infected, asymptomatically infected and removed individuals, respectively. We assumed that exposed people do not transmit the pathogen [28–30], and that severely infected individuals are not so critical as to be refrained from community activities due to hospitalization or isolation but carry a higher virus load. Therefore people in *I*_*s*_, *I*_*m*_ and *A* are all capable of contracting the disease but with different infectiousness levels. These levels were specified by *ρ*_*s*_ and *ρ*_*a*_, denoting an increased and a decreased infectiousness of severely and asymptomatically infectious individuals compared to mildly infected ones. A fraction *p*_*a*_ of exposed individuals was assumed to develop into asymptomatic infections, and among the rest, a further fraction *p*_*s*_ will develop into severe infections while the remaining become mildly infected. For the rest of the parameters, *N* is the constant total population size; *β* is the per contact transmission probability of mild cases; *σ* is the disease progression rate from latency to infectiousness with 1/*σ* being the mean incubation duration; and *γ*_*s*_, *γ*_*m*_ and *γ*_*a*_ are recovering rates of different types of cases with the reciprocals equal to the corresponding mean infectious periods. It was also assumed that the infection-acquired immunity lasts longer than the time period under study, so re-infection was not considered. Mitigation interventions, like quarantine, treatment, vaccination, etc., were not incorporated as we only wanted to investigate the early stage with little or no disease control in place. Other COVID-19 infection phases, such as pre-symptomatic stage, were not considered in order to keep the model simple and to focus on the particular characteristics of interest.

The basic reproduction number of system (2.1) was calculated using the next-generation matrix approach [31] as

2.2 $$R_{0}=(1-p_{a})p_{s}\frac{\rho_{s}\beta }{\gamma_{s}}+(1-p_{a})(1-p_{s})\frac{\beta }{\gamma_{m}}+p_{a}\frac{\rho_{a}\beta }{\gamma_{a}},$$

where the first term accounts for the average number of secondary infections coming from severe infections, the second term from mild infections and the last from asymptomatic infections. Through (2.2), *β* can be expressed in terms of $R_{0}$ and other parameters as

2.3 $$\beta =\frac{R_{0}}{\left[((1-p_{a})p_{s}\rho_{s}/\gamma_{s})+(\left(1-p_{a})(1-p_{s}\right)/\gamma_{m})+(\;p_{a}\rho_{a}/\gamma_{a})\right]}.$$

To incorporate under-reporting, we assumed that a fraction *r*_*s*_ of severe cases, a fraction *r*_*m*_ of mild cases and no asymptomatic cases were reported, which is often the case with a novel infection outbreak. The reporting rates *r*_*s*_ and *r*_*m*_ were fixed to be constants over time in light of the relatively short period of time considered in this study, though they may well vary in different stages of transmission. With these changes, we obtained a new system of equations (2.4) and (2.5) based on (2.1). This system is similar to the systems formulated in earlier studies [32–34]. In (2.4), *C*_*I*_ and *C*_*K*_ denote the true and reported cumulative number of cases, respectively. The derivation was completed by observing that *C*′_*I*_(*t*) = *σE*(*t*), and thus the equation of *E*(*t*) can be rewritten using the equation of *S*(*t*) as *E*′(*t*) = −*S*′(*t*) − *C*′_*I*_(*t*). Integrating both sides and using initial conditions *S*(0) = *N* and *C*_*I*_(0) = *E*(0) = 0 yields *S*(*t*)/*N* = 1 − ((*E*(*t*) + *C*_*I*_(*t*))/*N*).

2.4 $$\begin{array}{rl} \frac{\;dC_{I}}{\;dt} & =\sigma E,\\ \frac{\;dC_{K}}{\;dt} & =r_{m}(1-p_{a})(1-p_{s})\sigma E+r_{s}(1-p_{a})p_{s}\sigma E,\\ \frac{\;dE}{\;dt} & =\frac{R_{0}}{\left((1-p_{a})p_{s}\rho_{s}/\gamma_{s})+(\left(1-p_{a})(1-p_{s}\right)/\gamma_{m})+(\;p_{a}\rho_{a}/\gamma_{a}\right)}\\ & \;\times \left(1-\frac{E+C_{I}}{N}\right)(\rho_{s}I_{s}+I_{m}+\rho_{a}A)-\sigma E,\\ \frac{\;dI_{m}}{\;dt} & =(1-p_{a})(1-p_{s})\sigma E-\gamma_{m}I_{m},\\ \frac{\;dI_{s}}{\;dt} & =(1-p_{a})p_{s}\sigma E-\gamma_{s}I_{s}\\ \;\mathrm{and}\;\frac{\;dA}{\;dt} & =p_{a}\sigma E-\gamma_{a}A, \end{array}\}$$

with non-negative initial values

2.5 $$\begin{array}{rl} C_{I}(0) & =I_{m0}+I_{s0}+A_{0}+R,\;C_{K}(0)=C_{K0},\;E(0)=E_{0},\\ & I_{m}(0)=I_{m0},\;I_{s}(0)=I_{s0}\;\mathrm{and}\;A(0)=A_{0}. \end{array}\}$$

In (2.5), *R* is the number of cases that had already recovered before the start of official reporting and *C*_*K*0_ is the number of reported cumulative cases on the first day of reporting.

### 2.2. Data

We collected daily reported cases and cumulative reported cases of COVID-19 which are publicly available on Our World in Data [35]. Based on data availability, we considered 54 African countries: Algeria, Angola, Benin, Botswana, Burkina Faso, Burundi, Cameroon, Cape Verde, Central African Republic, Chad, Comoros, Congo, Cote d’Ivoire, Democratic Republic Congo, Djibouti, Egypt, Equatorial Guinea, Eritrea, Eswatini, Ethiopia, Gabon, Gambia, Ghana, Guinea, Guinea Bissau, Kenya, Lesotho, Liberia, Libya, Madagascar, Malawi, Mali, Mauritania, Mauritius, Morocco, Mozambique, Namibia, Niger, Nigeria, Rwanda, São Tomé and Príncipe, Senegal, Seychelles, Sierra Leone, Somalia, South Africa, South Sudan, Sudan, Tanzania, Togo, Tunisia, Uganda, Zambia and Zimbabwe. Each country’s total population size *N* in 2020 and the initial number of reported cases *C*_*K*0_ were also collected from the same source [35].

The first COVID-19 case was reported on different dates for each country and their reaction to the pandemic in terms of mitigation measures were also quite different. Hence, the initial phase of the COVID-19 outbreak considered in this study differs by country on the dates and numbers of data points included. The starting point was chosen to be the date on which the first case was reported, whereas the ending point was chosen to be the last date with Country Stringency Index ≤50 from Oxford COVID-19 Government Response Tracker [36]. Five out of 54 countries (9.3%) have missing Stringency Index data (Burundi, Comoros, Equatorial Guinea, Guinea Bissau, and S$\tilde{a}$o Tomé and Príncipe). Thirty-six countries (66.7%) have the duration of this phase shorter than 14 days, which means these countries either imposed their strict intervention strategies within two weeks of their first officially reported case or had begun to do so before the occurrence of their first case. To have enough data points for fitting and also taking into account the delayed compliance to government policies in reality, for countries with no index data or too short a period, a minimum of 14 days was used instead. All country-specific parameter values are summarized in the electronic supplementary material.

### 2.3. Fitting model to case data

The model (2.4) and (2.5) was fitted to reported cumulative cases and daily cases of COVID-19 for each country taken from Our World in Data [35] during the beginning of the epidemic. The number of daily reported cases is denoted by *I*_*K*_ and was calculated by our model as

2.6 $$I_{K}=r_{m}(1-p_{a})(1-p_{s})\sigma E+r_{s}(1-p_{a})p_{s}\sigma E.$$

We used Markov chain Monte Carlo method (MCMC) with delayed rejection adaptive metropolis (DRAM) algorithm with non-informative uniform priors and sum of squares (SS) given in (2.7) to fit the model, where *t*_data_ are the chosen time points for each country; $C_{K,\mathrm{data}}$ and $I_{K,m}$ are observed cumulative and daily cases corresponding to *t*_data_. The algorithm was implemented through MCMC toolbox for MATLAB [37] with 5000 iterations and adapted proposal every 500 iterations for each country. Since milder cases are usually less transmissible due to fewer coughs [38], we assumed a decreased transmissibility of asymptomatic cases (*ρ*_*a*_ ≤ 1) but an increased transmissibility of severe cases (1 ≤ *ρ*_*s*_ ≤ 1.5). The corresponding values for fixed parameters and the initial values, minimum and maximum values used for MCMC simulations for estimated parameters are listed in table 1.

2.7 $$SS=\sum_{t\in t_{\mathrm{data}}}{\left(C_{K}(t;\theta )-{\left(C_{K,\mathrm{data}}\right)}_{t}\right)}^{2}+{\left(I_{K}(t;\theta )-{\left(I_{K,\mathrm{data}}\right)}_{t}\right)}^{2}.$$

**Table 1.** Table of fixed parameters and unfixed parameters with initial values, minimum and maximum values for MCMC run.

symbol	description	initial value	min.	max.
$R_{0}$	basic reproduction number	2.37 [39]	1	3.9 [40,41]
*ρ*_*s*_	relative infectiousness of severely infectives compared to mildly infectives	1 [42]	1	1.5
*ρ*_*a*_	relative infectiousness of asymptomatically infectives compared to mildly infectives	0.55 [27]	0	1
*p*_*s*_	proportion of severe infections among symptomatic infections	0.202 [42]	0	1
*p*_*a*_	proportion of asymptomatic infections among all infections	0.46 [40]	0	1
*r*_*m*_	report rate of mild infections	0.25 [43,44]	0	1
*r*_*s*_	report rate of severe infections	0.5	0	1
*E*_0_	initial exposed population size	*C*_*K*0_	*C*_*K*0_	N
*I*_*m*0_	initial mildly infected population size	*C*_*K*0_	*C*_*K*0_	N
*I*_*s*0_	initial severely infected population size	*C*_*K*0_	*C*_*K*0_	N
*A*_0_	initial asymptomatically infected population size	*C*_*K*0_	*C*_*K*0_	N
*R*	initial recovered population size	0	0	N
*σ*	progression rate from exposed to infectious	1/5.1 [45]		
*γ*_*a*_	recovery rate for asymptomatic infectives	1/5.88 [42]		
*γ*_*s*_ = *γ*_*m*_	recovery rate for symptomatic infectives	1/4.2 [42]		
*N*	total population size	country specific		
*C*_*K*0_	initial reported cumulative infections	country specific		

Posterior distributions for the true basic reproduction number, report rate of mild and severe cases, fraction of severe and asymptomatic cases, and relative infectiousness of severe and asymptomatically infectious individuals to mild infectives along with initial values were then estimated.

## 3. Results

The densities of posterior distributions of the estimated parameters are given in the electronic supplementary material. From these, we further calculated the mean estimates of reproduction number of reported cases (denoted by $R_{0K}$), fraction of mild cases among all cases (denoted by ${\tilde{\;p}}_{m}$), fraction of severe cases among all cases (denoted by ${\tilde{\;p}}_{s}$) and overall report rate (denoted by *r*_all_) as in (3.1).

3.1 $$\begin{array}{rl} & R_{0K}=(1-p_{a})p_{s}\frac{\rho_{s}\beta }{\gamma_{s}}+r_{m}(1-p_{a})(1-p_{s})\frac{\beta }{\gamma_{m}},\\ & {\tilde{\;p}}_{m}=(1-p_{a})(1-p_{s}),\\ & {\tilde{\;p}}_{s}=(1-p_{a})p_{s}\\ \;\mathrm{and}\; & r_{\mathrm{all}}=r_{s}(1-p_{a})p_{s}+r_{m}(1-p_{a})(1-p_{s}). \end{array}\}$$

Table 2 summarizes all mean values of these parameters for all countries. The countries are grouped into regions for organizational purpose.

**Table 2.** Mean estimation of parameters for each country.

country	$R_{0}$	$R_{K0}$	*ρ*_*s*_	*ρ*_*a*_	${\tilde{\;p}}_{s}\;(\%)$	$\;p_{m}\;(\%)$	$\;p_{a}\;(\%)$	$r_{m}\;(\%)$	$r_{s}\;(\%)$	$r_{\mathrm{all}}\;(\%)$	*E*_0_	*I*_*m*0_	*I*_*s*0_	*A*_0_	*R*
*central*															
Burundi	2.26	0.01	1.25	0.55	0	30.33	69.67	0.88	25.70	0.27	763.75	14.32	20.16	9.65	0
Cameroon	2.96	0.06	1.26	0.10	0.48	46.22	53.30	1.63	62.23	1.06	6.59	19.12	2.27	805.66	0
Central African Republic	1.40	0.01	1.25	0.56	0.74	7.16	92.11	3.22	49.88	0.60	470.72	7.06	1.68	30.93	0
Chad	1.63	0.15	1.27	0.61	7.96	11.32	80.73	35.47	40.33	7.22	13.64	5.96	9.33	1.51	0
Congo	1.41	0.01	1.17	0.65	0.52	24.90	74.58	1.98	25.76	0.63	644.31	10.67	5.31	4.41	0
DR Congo	2.77	0.07	1.25	0.30	2	59.63	38.37	2.30	27.13	1.91	57.12	7.41	4.38	917.75	0
Equatorial Guinea	1.32	0.08	1.21	0.67	5.14	25.25	69.61	11.86	48.75	5.50	55.83	63.16	31.60	2.86	0
Gabon	1.58	0.16	1.26	0.52	5.94	27.46	66.60	19.08	43.93	7.85	3.49	18.16	12.36	14.02	0
São Tomé and Príncipe	2.07	0	1.27	0.57	0	34.67	65.33	0.05	12.16	0.02	2751.79	30.44	10.77	11.16	0
*eastern*															
Comoros	1.32	0.12	1.24	0.65	5.40	10.04	84.56	40.52	61.21	7.38	35.41	44.87	31.36	1.76	0
Djibouti	2.38	0.33	1.25	0.21	11.52	36.57	51.91	12.26	32.28	8.20	21.25	3.63	2.13	298.14	0
Eritrea	2.21	0.17	1.22	0.40	4.39	23.74	71.87	14.36	36.60	5.02	12.92	1.78	2.63	258.46	0
Ethiopia	1.26	0.05	1.23	0.65	3.27	5.37	91.36	43.06	40.71	3.64	171.39	34.16	27.83	2.43	0
Kenya	2.78	0.45	1.27	0.14	2.31	24.10	73.58	22.22	46.40	6.43	42.31	2.43	2.38	225.53	0
Madagascar	1.54	0.32	1.25	0.53	12.18	26.65	61.16	33.39	59.67	16.17	19.14	72.62	77	15.78	0
Mauritius	1.43	0.15	1.24	0.75	6.15	41.97	51.88	16.50	54.30	10.26	8.60	4.40	6.34	1057.52	0
Rwanda	1.99	0.52	1.23	0.51	11.80	41.51	46.69	37.73	54.50	22.09	20.33	2.05	1.40	167.83	0
Seychelles	1.28	0	1.19	0.51	0.02	10.82	89.15	1.94	28.79	0.22	1839.29	4.08	5.05	2.73	63 940
Somalia	2.36	0.11	1.27	0.34	4.21	40.26	55.53	6.76	14.63	3.34	3.51	1.79	1.92	26.06	0
South Sudan	1.21	0	1.23	0.61	0.05	4.25	95.70	0.79	40.64	0.06	5540.67	4.04	11.83	8.96	0
Sudan	2.18	0.23	1.29	0.53	8.31	16.72	74.97	19.68	51.41	7.56	2.30	2.91	2.68	2.34	0
Tanzania	1.16	0	1.25	0.57	0.05	6.41	93.54	4.92	20.71	0.33	3248.18	14.90	11.65	7.22	0
Uganda	1.23	0.12	1.26	0.65	8.59	10.70	80.71	45.49	43.78	8.63	199.23	119.81	160.23	2	0
*northern*															
Algeria	3.39	0.46	1.26	0.23	7.31	53.96	38.72	13.92	31.17	9.79	6.57	2.23	3.08	25.56	0
Egypt	2.80	0.37	1.27	0.29	3.69	25.46	70.85	21.71	44.67	7.18	4.47	2.02	1.44	28.58	0
Libya	1.43	0.50	1.24	0.56	39.45	21.24	39.31	46.31	52.16	30.41	3.17	16.68	12.16	1.54	0
Mauritania	2.35	0.03	1.25	0.49	2.02	46.77	51.21	0	45.08	0.91	109.47	10.72	9.25	3.06	0
Morocco	2.90	0.16	1.23	0.05	5.79	46.30	47.91	2.70	26.38	2.78	20.53	10.20	8.47	794.37	0
Tunisia	2.33	0.23	1.23	0.13	2.13	46.03	51.84	10.08	36.80	5.42	22.67	2.40	17.55	281.21	0
*southern*															
Angola	1.55	0.06	1.28	0.29	1.62	21.98	76.40	4.72	51.51	1.87	184.88	1.76	3.05	108.66	0
Botswana	1.99	0.06	1.28	0.40	1.69	50.89	47.42	3.57	28.05	2.29	73.43	9.40	19.93	274.36	0
Eswatini	2.71	0.17	1.27	0.09	9.07	49.60	41.33	3.16	23.10	3.66	2.83	2.27	2.25	216.23	0
Lesotho	2.65	0.06	1.25	0.56	1.62	25.66	72.72	4.80	37.51	1.84	2.49	4.39	4.83	13.76	0
Malawi	1.55	0.16	1.22	0.55	10.36	17.44	72.20	18.99	41.77	7.64	55.60	8.05	5.07	59.88	0
Mozambique	1.25	0	1.25	0.62	0.01	6.40	93.59	0.86	24.28	0.06	14566.76	1.44	3.12	5.73	0
Namibia	2.22	0.14	1.25	0.48	13.14	21.99	64.87	9.69	19.21	4.65	3.88	8.54	5.30	49.27	0
South Africa	2.94	0.68	1.27	0.06	13.42	39.68	46.90	18.78	39.03	12.69	45.82	5.18	4.48	846.23	0
Zambia	1.12	0.08	1.24	0.65	2.17	12.48	85.35	45.48	47.84	6.71	125.65	64.56	101.03	4.79	0
Zimbabwe	1.48	0.01	1.25	0.48	0	45.31	54.69	0.99	50.79	0.45	1220.56	6.25	3.48	38.74	0
*western*															
Benin	1.33	0.10	1.26	0.46	5.60	5.17	89.24	47.83	40.72	4.75	41.57	32.57	15.88	1.76	0
Burkina Faso	3.03	0.21	1.28	0.17	8.21	42.96	48.83	3.99	27.64	3.98	91.22	4.81	8.49	1323.52	0
Cape Verde	1.21	0.01	1.24	0.53	0.01	33.07	66.92	1.35	47.90	0.45	965.60	9.20	11.32	5.29	0
Cote d’Ivoire	2.77	0.30	1.26	0.35	7.82	34.95	57.23	11.49	39.28	7.09	43.21	2.84	3.81	251.61	0
Gambia	1.74	0.25	1.28	0.57	17.66	28.53	53.81	12.37	43.46	11.21	4.64	4.86	5.38	2.16	0
Ghana	3.09	0.28	1.27	0.36	7.37	31.71	60.92	6.68	47.35	5.61	75.46	14.18	8.18	831.06	0
Guinea	2.25	0.10	1.26	0.50	4.47	25	70.53	4.34	43.63	3.04	1.43	6.06	4.56	53.35	0
Guinea Bissau	2.56	0.57	1.27	0.11	6.54	31.29	62.17	24.07	41.60	10.25	19.61	6.79	3.52	102.81	0
Liberia	1.23	0	1.23	0.56	0.15	5.11	94.73	1.02	36.45	0.11	2091.46	3.59	2.08	1.85	0
Mali	1.29	0.02	1.23	0.63	0.58	11.45	87.97	7.13	44.57	1.08	2772.71	186.49	185.23	8.79	0
Niger	2.88	0.08	1.25	0.17	1.94	34.27	63.79	1.44	36.63	1.21	175.65	15.29	18.22	4855.52	0
Nigeria	3.64	0.17	1.21	0.17	8.88	55.22	35.90	3.11	16.08	3.15	3.85	1.67	1.40	16.48	0
Senegal	2.61	0.48	1.24	0.20	8.09	36.91	55	21.35	37.34	10.90	8.70	3.67	4.10	72.50	0
Sierra Leone	1.34	0.07	1.22	0.57	2.93	25.95	71.12	13.03	34.64	4.39	125.55	14.72	14.25	1.89	0
Togo	1.52	0	1.27	0.60	0.02	2.60	97.38	0.66	5.33	0.02	588.10	5.13	2.61	7.89	0
all countries															
mean	2.02	0.17	1.25	0.44	5.46	27.80	66.74	13.74	38.21	5.37	729.43	17.74	17.33	262.28	1184.07
s.d.	0.70	0.17	0.02	0.20	6.40	15.39	17.28	14.45	12.71	5.71	2180.24	31.91	35.33	711.13	8701.13

**Figure 2.**
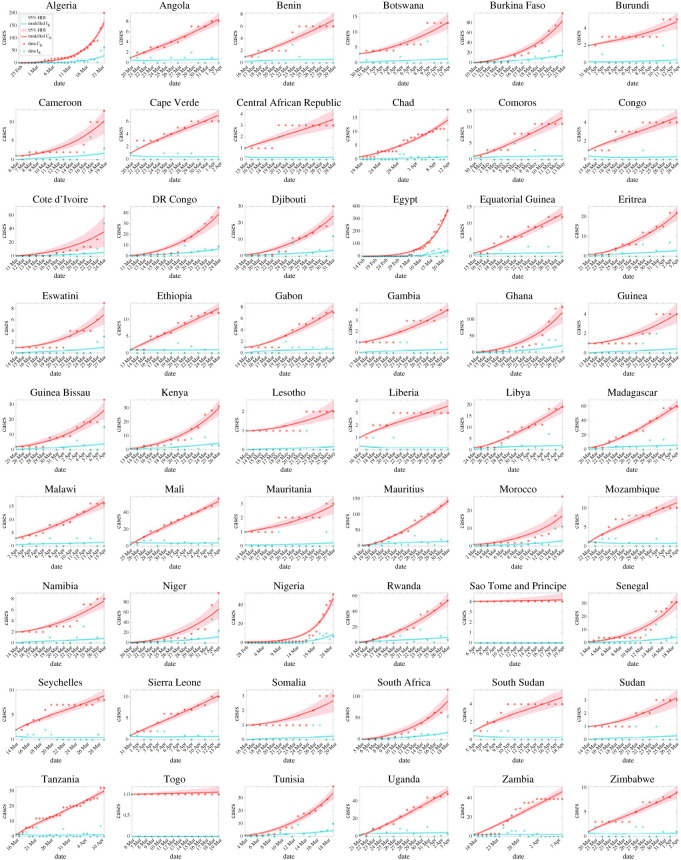
Mean model prediction and data for cumulative reported cases (red line and red circles) and daily reported cases (green line and green circles) in each country for the initial time period with no major mitigation at national level. Coloured areas show 95% high density intervals (HDI) of prediction.

Cumulative and daily reported cases together with the predicted values obtained from our model are presented in figure 2. The red and green circles are the cumulative and daily reported cases of COVID-19, respectively, while the mean predicted values are shown in red and green lines and the coloured bands represent the 95% high-density intervals (HDI) of prediction. Judging from figure 2, the mean fits of reported cumulative cases *C*_*K*_ and new cases *I*_*K*_ to data are generally good for each country. Notice that in this initial phase, reported cases grow exceptionally fast in Egypt, Algeria, South Africa, Ghana, Mauritius, Burkina Faso and Rwanda, while case numbers were not updated and stayed at a low level for Togo and São Tomé and Príncipe. In countries like Benin, Botswana, Burundi, Cabo Verde, Central African Republic, Comoros, Congo, Eswatini, Gambia, Guinea, Lesotho, Liberia and Mauritania, plots of cumulative cases demonstrated a stepwise shape implying a possibility of discontinued and unstable testing or reporting practice.

**Figure 3.**
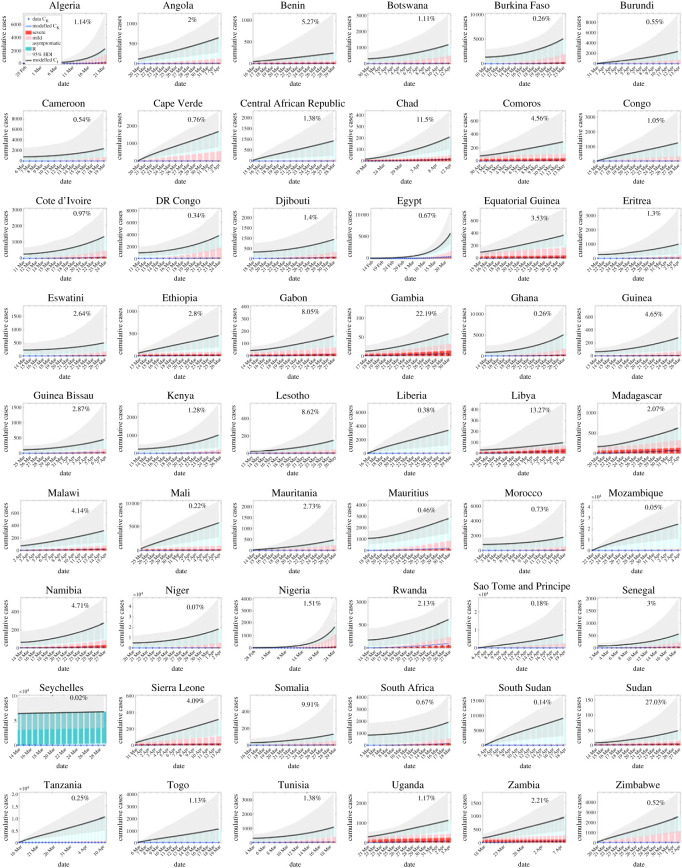
Estimated mean true cumulative cases (black line) with modelled and data of reported cumulative cases (blue line and blue star) in each country for the initial time period with no major mitigation at national level. Coloured bars show the percentage of severe (darker red), mild (lighter red), asymptomatic (lighter green) and already recovered cases before the reporting started (darker green). Grey shaded areas show 95% high density intervals (HDI).

In figure 3, the predicted mean true cumulative cases *C*_*I*_ (black line) with 95% HDI (shaded area) are plotted as opposed to reported cumulative cases *C*_*K*_ (blue line for modelled values and blue stars for data). For each day’s estimated true cumulative cases, they were further decomposed into severe cases (darker red bars), mild cases (lighter red bars), asymptomatic cases (lighter green bars) and already recovered cases before the official reporting started (darker green bars). The number at the top right corner indicates the percentage of cases reported cumulatively by the end of this initial phase. Throughout countries, the predicted true numbers of cumulative cases are high above the reported ones in all times, with Sudan and Gambia reporting collectively most (27.03% and 22.19%, respectively) while most countries reported less than 5%. Severe cases made up only a very small fraction of all predicted cases, but are of a more significant proportion in Libya, Gambia, Madagascar, Uganda, etc. Meanwhile apparently asymptomatic infections are the majority. Extraordinarily, in Seychelles the already recovered cases constitute the largest portion.

**Figure 4.**
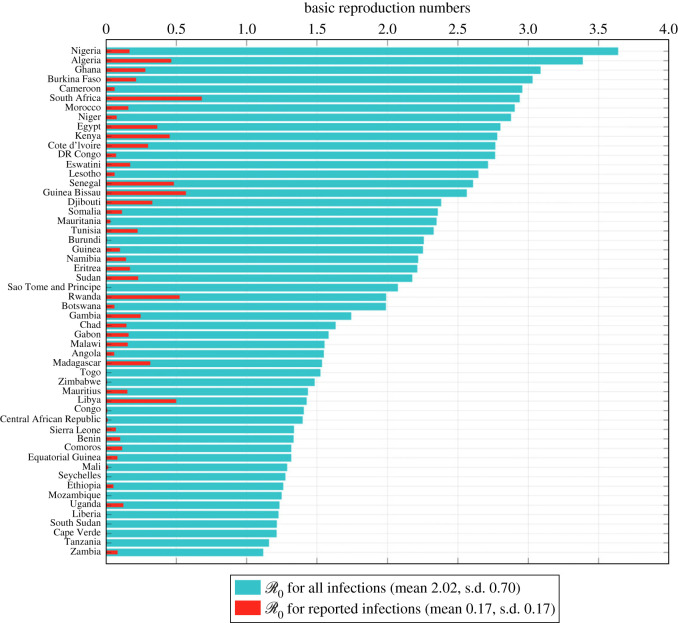
Mean basic reproduction number $R_{0}$ for all infections (green bars) and mean basic reproduction number $R_{0K}$ for observed cases (red bars). The meta mean over all countries of $R_{0}$ is 2.02 (s.d. 0.70) and 0.17 (s.d. 0.17) of $R_{0K}$.

We estimated that the mean basic reproduction number $R_{0}$ is 2.02 (s.d. 0.70), with a range between 1.12 (Zambia) and 3.64 (Nigeria), whereas the mean basic reproduction number for observed cases $R_{K0}$ was estimated to be 0.17 (s.d. 0.17), with a range between 0 (São Tomé and Príncipe, Seychelles, Tanzania, South Sudan, Mozambique, Liberia, Togo) and 0.68 (South Africa). The true and basic reproduction numbers for observed cases for all countries are plotted in figure 4. Ubiquitously, $R_{K0}$ is less than $R_{0}$ under the assumption that asymptomatic cases were not reported and only a proportion of mild cases were reported. Countries with high $R_{0}$ may have surprisingly low $R_{0K}$ like Nigeria, Cameroon, Niger, Democratic Republic Congo, Lesotho, etc., due to a majority of infections being asymptomatic or mild and/or a very low report ratio of mild infections. Moreover, countries with $R_{0K}<1$ suggesting no outbreak within the territory may actually have true $R_{0}$ way higher than 1, indicating a definite outbreak. Thus, neglecting under-reporting will likely cause a large underestimation of the basic reproduction number and furthermore the severity and magnitude of localized epidemic.

**Figure 5.**
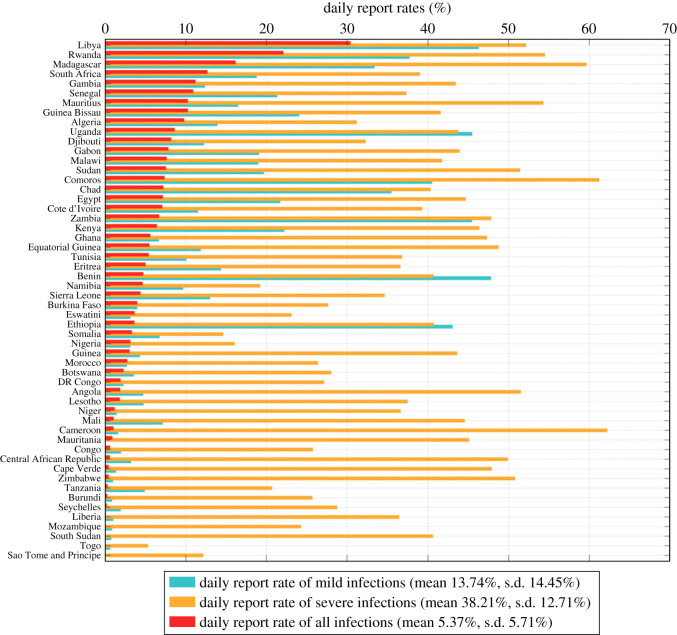
Reporting rates *r*_*m*_ of mild infections (green bars), *r*_*s*_ of severe infections (yellow bars) and overall reporting rates *r*_all_ of all infections (red bars). The meta mean over all countries is 13.74% (s.d. 14.45%) of *r*_*m*_, 38.21% (s.d. 12.71%) of *r*_*s*_ and 5.37% (s.d. 5.71)% of *r*_all_.

**Figure 6.**
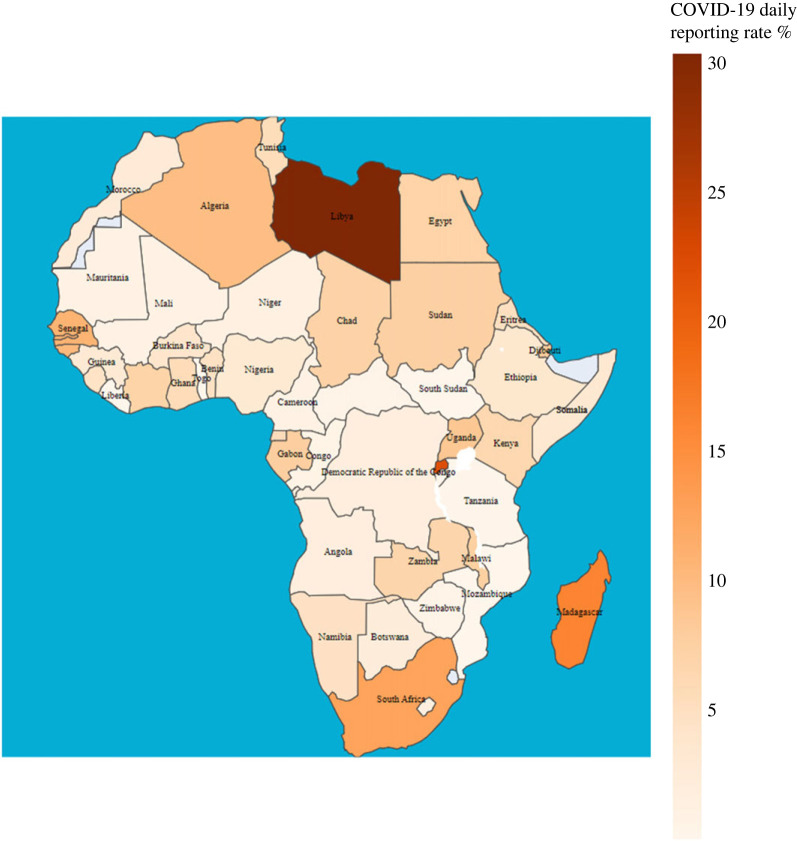
Estimated overall COVID-19 case reporting rate *r*_all_ for 54 countries in Africa.

We estimated that the mean overall daily report rate *r*_all_ is 5.37% (s.d. 5.71%) among all countries, with the highest 30.41% in Libya and the lowest 0.02% in São Tomé and Príncipe. The estimated mean report ratio of severe infections *r*_*s*_ was 38.21% (s.d. 12.71%) with the highest 62.23% in Cameroon and the lowest 5.33% in Togo, and the estimated mean report ratio of mild infections *r*_*m*_ was 13.74% (s.d. 14.45%) with the highest 47.83% in Benin and the lowest 0% in Mauritania. Reporting ratios of all kinds for all countries are plotted in figure 5. Figure 6 summarizes the overall report rate across countries. Notice that in most countries, the severe infection report rate was higher than mild infection except in Uganda, Benin and Ethiopia, although the ratios did not differ much. Also, countries with a relative high/low severe infection report rate tend to have high/low overall report rate, but those with very high mild case report rate may still exhibit low overall rate due to a high percentage of asymptomatic case constitution like Cameroon, Zimbabwe and the Central African Republic.

**Figure 7.**
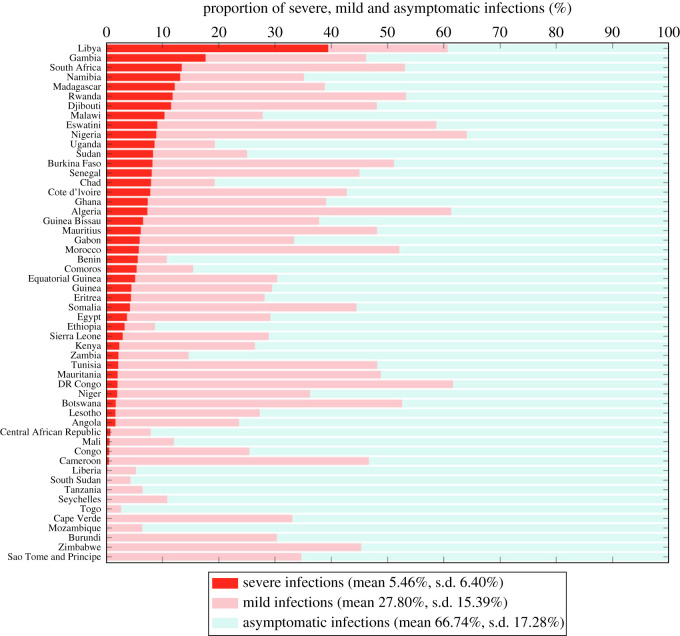
Proportions of severe ${\tilde{p}}_{s}$ (darker red bars), mild ${\tilde{\;p}}_{m}$ (lighter red bars) and asymptomatic *p*_*a*_ (green bars) infections. The meta means over all countries of them are 5.46% (s.d. 6.40%), 27.80% (s.d. 15.39%) and 66.74% (s.d. 17.28%) respectively.

**Figure 8.**
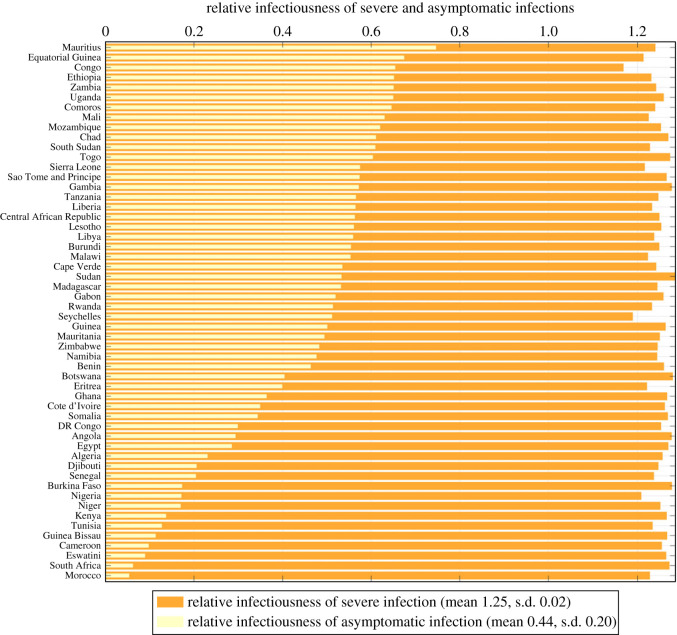
Relative infectiousness of severe *ρ*_*s*_ (orange bars) and asymptomatic infections *ρ*_*a*_ (yellow bars). The meta means over all countries of them are 1.25 (s.d. 0.02) and 0.44 (s.d. 0.20) respectively.

As for proportions of different types of infections, the mean estimates were 5.46% (s.d. 6.40%) for severe infections, 27.80% (s.d. 15.39%) for mild ones and 66.74% (s.d. 17.28%) for asymptomatic ones. Proportions for all countries are plotted in figure 7. This figure suggests that subclinical infections generally make up the majority of all COVID-19 cases, but specific proportions vary from location to location. The mean estimations of relative infectiousness of severely and asymptomatically infected individuals were 1.25 (s.d. 0.02) and 0.44 (s.d. 0.20) with values for all countries plotted in figure 8. The variation of those for severe infection was comparatively small among countries, suggesting a more intrinsic characteristic to SARS-CoV-2 rather than one subject to demographic or geological change. The relative infectiousness of asymptomatic infection was lower in a small number of countries like Morocco, South Africa, Eswatini, Cameroon, Guinea Bissau, etc.

## 4. Discussion

By fitting a simple deterministic mathematical model distinguishing various levels of infections and accounting for level-specific reporting rates to case data for each African country, we estimated crucial epidemiological parameters describing and characterizing the COVID-19 early outbreak and thereby provided a country-wise epidemic profile for this continent. Our findings suggest that the basic reproduction numbers were much higher than when only clinically reported cases were taken into consideration and the average overall case reporting rate was rather low in this early stage of localized outbreak.

Africa is unique in its younger population structure. Younger people are shown to be less susceptible to SARS-CoV-2 [13] implying limited spread and less severity in epidemic magnitude [17,46]. They are also found to be less likely of showing clinical symptoms compared with senior people [13], which further suggests a higher possibility of lower detection rates in countries mostly made up of young population like in Africa. Additionally, in an early stage of a novel transmissible disease outbreak, confirmation definitions and surveillance systems were not yet consistently established; meanwhile given the lack of public health resources like testing and cultural stigmatization in Africa, the overall ascertainment rate of SARS-CoV-2 can be even lower. Using a mathematical model with essential epidemiological characteristics of the pathogen, together with available observed data to have an estimation of the under-ascertainment rate and the true epidemic magnitude, can therefore help to some extent unveil the hidden infections and thereby better evaluate the disease severity and cope with it more appropriately.

Our estimation of the mean overall reporting rate of 5.37% for Africa is consistent with the estimation in the early stage of COVID-19 in other countries. In Wuhan, China, where the very first SARS-CoV-2 cases were reported, the case ascertainment rate was estimated to be 5.0% (95% CI, 3.6–7.4) [47]. Another estimation for 375 cities in China before the initial travel restriction of the documentation rate was 14% (95% CI, 10–18) [27]. In a modelling study in 92 nations across the world, the estimated ratio of true to reported cumulative cases through the end of 2020 was 7.03 (10–90% percentile, 3.2–18), corresponding to a cumulative reporting ratio of 14.22% (10–90% percentile, 5.56–31.25) [24]. It is also consistent with seroprevalence study: less than 1% of all infected cases were reported according to an autumn 2020 seroprevalence study in Juba in South Sudan [48], compared to our estimation of overall reporting rate of 0.06% for South Sudan in the beginning stage. Our estimation that on average, more than half (66.74%) of all infections were asymptomatic is in agreement with the asymptomatic infection rate estimate of 46% (95% CI, 18.48–73.60) [49], 50% in 92 countries [24] and 40–45% in cohorts of various locations [50]. And similarly, the asymptomatic ratio displays small variations across nations [24]. Our estimation of the relative infectiousness of asymptomatic population of 44% also roughly agrees with the estimation of 55% (95% CI, 46–62) in China [27]. Nonetheless, our more detailed model provided extensive estimates as to the reporting ratios of various levels of infections, detailed proportions of the infections among all cases and their corresponding relative infectiousness. Noting some of the quantities reflect intrinsic characteristics of SARS-CoV-2, these estimations could be used for understanding or modelling outside Africa too.

The nation-specific overall COVID-19 case reporting rates shown in figure 6 suggests generally higher rates in northern and southern Africa than central Africa. This result agrees principally with the knowledge of the public healthcare system quality in the continent: public expenditure on healthcare among total healthcare expenditure for 2019 [51] and its share of GDP [52] both show that northern and southern Africa spend more than the central region, and moreover Libya is among the top African countries with most nurses and physicians per 1000 population [53,54]. However, it is left to further study to identify the key factors determining these differences in case reporting in the beginning of a novel outbreak within Africa.

The reporting ratio was assumed to be constant throughout the initial phase of the outbreak in our study, which is acceptable due to the short average duration of this phase. But it needs acknowledgement that testing and reporting practices greatly vary as the disease outbreak goes on. Therefore, for a better estimation of under-reporting for longer period, time-dependent rates are necessary, as in studies using also mechanistic models [26,27]. Inversion method can also be of use for such estimation without the need to assume a specific functional form for ascertainment rate over time. Techniques other than mechanistic mathematical modelling had also been used to estimate the level of under-ascertainment, such as ratio of baseline case fatality rate (CFR) and delay-adjusted CFR [55], which gives a time-dependent under-ascertainment rate rather than a constant one.

One major limitation to this study was the short duration and great data uncertainties within this period. African countries implemented their public health and social measures (PHSMs) very early: 36 out of 50 (72%) countries implemented their first stringent PHSM a mean of 15 days before reporting their first case and the other 14 countries implemented a mean of 9 days after their first case [56]. Thus, the initial stages were generally quite short for all countries considered here. Although there is an advantage in studying only the initial stage of COVID-19 outbreak, that there were no major mitigation interventions yet, allowing simpler epidemiological models, there is also great disadvantage in the reliability of the data. As mentioned before, case reporting and data collecting were not yet well established, making the data a relatively poor representation of the routine surveillance and reporting practice of each country.

Few studies have tried to provide a complete COVID-19 epidemic profile for Africa. Our work here might give some insight into the characteristics of the transmission of SARS-CoV-2 in this continent and a comparison among nations. Our more detailed model might also provide an extended estimation of under-ascertainment of cases, thereby offering material for a more accurate assessment of the true epidemic size in Africa. Nevertheless, the estimated low reporting rates in Africa suggested a clear need for improved reporting and surveillance systems in these countries, with a special emphasis on central Africa.

## Data Availability

The datasets used for this study are publicly available on Our World in Data (https://ourworldindata.org/covid-cases). The data are provided in electronic supplementary material [57].
